# An Innovative Hyperbaric Hypothermic Machine Perfusion Protects the Liver from Experimental Preservation Injury

**DOI:** 10.1100/2012/573410

**Published:** 2012-04-19

**Authors:** Ferdinando A. Giannone, Davide Treré, Marco Domenicali, Ignazio Grattagliano, Alessandra Baracca, Gianluca Sgarbi, Caterina Maggioli, Pasquale Longobardi, Giancarlo Solaini, Massimo Derenzini, Mauro Bernardi, Paolo Caraceni

**Affiliations:** ^1^Department of Clinical Medicine, Alma Mater Studiorum University of Bologna, 40138 Bologna, Italy; ^2^Center for Applied Biomedical Research (C.R.B.A.), Alma Mater Studiorum University of Bologna, 40138 Bologna, Italy; ^3^Department of Experimental Pathology, Alma Mater Studiorum University of Bologna, 40126 Bologna, Italy; ^4^Department of Internal and Public Medicine, University of Bari, 70126 Bari, Italy; ^5^Department of Biochemistry, Alma Mater Studiorum University of Bologna, 40126 Bologna, Italy; ^6^Centro Iperbarico s.r.l. Ravenna, 48124 Ravenna, Italy

## Abstract

*Purpose*. Hypothermic machine perfusion systems seem more effective than the current static storage to prevent cold ischemic liver injury. Thus, we test an innovative hyperbaric hypothermic machine perfusion (HHMP), which combines hyperbaric oxygenation of the preservation solution and continuous perfusion of the graft. *Methods*. Rat livers were preserved with Celsior solution according to 4 different modalities: *normobaric static preservation*; *hyperbaric static preservation* at 2 atmosphere absolute (ATA); *normobaric dynamic preservation*, with continuous perfusion; *hyperbaric dynamic preservation*, with continuous perfusion at 2 ATA. After 24 h cold preservation, we assessed different parameters. *Results*. Compared to baseline, livers preserved with the current static storage showed severe ultrastructural damage, glycogen depletion and an increased oxidative stress. Normobaric perfused livers showed improved hepatocyte ultrastructure and ameliorated glycogen stores, but they still suffered a significant oxidative damage. The addition of hyperbaric oxygen produces an extra benefit by improving oxidative injury and by inducing endothelial NO synthase (eNOS) gene expression. *Conclusions.* Preservation by means of the present innovative HHMP reduced the liver injury occurring after the current static cold storage by lowering glycogen depletion and oxidative damage. Interestingly, only the use of hyperbaric oxygen was associated to a blunted oxidative stress and an increased eNOS gene expression.

## 1. Introduction

The prevention of preservation injury is crucial to accomplish the early recovery of cellular metabolism after transplantation and to avoid graft dysfunction. To bridge the timespan from the harvesting to the implantation in the recipient, the livers are stored in preservation solutions on melting ice at 0–4°C, allowing a safe preservation up to 12 h [1]. A cold ischemia longer than 12 h still implies a greater risk of graft dysfunction. Furthermore, the rising demand for transplantation triggers the use of the so-called extended criteria donors, which tolerate a period of cold ischemia even shorter than 12 hours [2].

Alteration of the energy metabolism is an important feature of preservation injury [3]. Hypothermia at 0–4°C is a key factor for organ preservation by reducing the cellular metabolic activity about 90–95% [4]. However, even at this low temperature, metabolism still requires 0.27 mol oxygen/min/g of liver [4], which is not provided by the current static storage. Oxygen is mainly taken up by mitochondria to allow the synthesis of adenosine triphosphate (ATP); therefore, the lack of oxygen results in failure of the respiratory chain and ATP can solely be generated through the anaerobic glycolysis. Once the cellular glycogen stores are consumed, ATP depletion rapidly ensues leading to a series of events which eventually cause irreversible cell injury and death [5].

In the last decade, experimental studies have shown that continuous perfusion of the liver during preservation can improve graft viability and challenge the limits of the current static storage [6–9]. However, several issues need to be clarified before the hypothermic machine perfusion (HMP) will reach the clinical application, including the type of preservation solution, the characteristics of perfusion dynamics, and the modalities of oxygen supply [4, 9]. Regarding the latter issue, oxygenation of the solution appears to be a typical double-edge condition: the oxygen supplied during cold ischemia should guarantee the ATP synthesis for the residual metabolic activity, but, at the same time, it might favor the generation of reactive oxygen species (ROS) leading to the exacerbation of cellular injury.

Treatment with hyperbaric oxygen is a method that employs exposure to 100% oxygen at a pressure above 1 atmosphere absolute (1 ATA) to promote tissue hyperoxygenation. In the setting of liver diseases, hyperbaric oxygen has been used to treat hepatic artery thrombosis after transplantation and acute liver failure due to carbon tetrachloride intoxication [10, 11]. However, experimental data indicate that hyperbaric oxygen is also capable to reduce hepatic warm ischemia-reperfusion injury by interfering with several pathogenic mechanisms [12–14]. It has been proposed that hyperbaric oxygen counteracts neutrophil adhesion within the microvasculature by downregulating the expression of endothelial cell adhesion molecules, enhance nitric oxide (NO) production by increasing the expression of endothelial NO synthase (eNOS), prevents lipid peroxidation, and ameliorates the mitochondrial oxidative phosphorylation capacity [12–14]. Finally, Ijichi et al. [15] have recently showed that hyperbaric oxygen applied to the current static storage ameliorates preservation injury and ATP depletion in rat livers.

Based on these observations, we aimed to determine whether an innovative hyperbaric hypothermic machine perfusion (HHMP) that combines the oxygenation of the preservation solution with hyperbaric oxygen to the continuous perfusion of the graft improves rat liver preservation injury.

## 2. Materials and Methods

### 2.1. Hyperbaric Hypothermic Machine Perfusion (HHMP)

The HHMP, designed and patented by “Centro Iperbarico s.r.l.,” Ravenna, Italy, consists of a hyperbaric container where the organ is stored, totally immersed in the preservation solution, with a residual free volume in the upper part containing a gas mixture of 95% O_2_ and 5% CO_2_. The hyperbaric chamber is enclosed into a conditioning system, which allows the exact regulation and control of the temperature and pressure (ranging form 0 to 2.5 ATA). The perfusion of the organ with the preservation solution throughout the portal vein is guaranteed by a peristaltic pump (Gilson Minupulse, Villiers Le Bel, France) located outside the hyperbaric chamber. A second peristaltic pump is used to generate a continuous movement of the preservation solution aiming to enhance oxygen diffusion within the preservation solution. Contrary to other HMP devices, which have been criticized for their complicated logistics, the HHMP has been designed to be easily carried by two persons allowing the possibility to start the intervention immediately after procurement and not only in the recipient's transplant centre.

### 2.2. Experimental Design

Fed Sprague-Dawley rats (Charles-River Laboratories, Calco, Italy), weighing 250–300 grams, were anesthetized (Zoletil-100, Virbac, France) between 9:00 and 10:00 a.m. and the abdomen opened with a midline incision. Afterwards, heparin 500 IU/liter was injected through the infrahepatic vena cava and the portal vein cannulated with a 16 G angiocath. The liver was then flushed out with 20 mL of cold Celsior solution, immediately explanted and flushed out again with 30 mL of cold Celsior solution. The livers were finally assigned to the following experimental groups:

1. *baseline controls*: liver tissue samples were immediately collected for the ultrastructural, histological, and biochemical analysis;
2. *normobaric static preservation*: livers were kept at 4°C in the HHMP under normobaric conditions (1 ATA) immersed in the Celsior solution for 24 h. This group mimics the current static storage used in clinical transplantation;
3. *hyperbaric static preservation*: livers were kept at 4°C in the HHMP under hyperbaric conditions (2 ATA) immersed in the Celsior solution for 24 h. Compression and decompression were carried out progressively at a rate of 0.2 ATA/min;
4. *normobaric dynamic preservation*: livers were kept at 4°C in the HHMP under normobaric conditions (1 ATA) and continuously perfused with Celsior solution at 1 mL/min/g liver for 24 h;
5. *hyperbaric dynamic preservation*: livers were kept at 4°C in the HHMP under hyperbaric conditions (2 ATA) and continuously perfused with Celsior solution at 1 mL/min/g liver for 24 h. Compression and decompression were carried out progressively at a rate of 0.2 ATA/min.

After 24 h of cold preservation, tissue samples were immediately collected for the ultrastructural, histological, and biochemical analysis. This preservation time was chosen based on preliminary experiments showing that liver damage increased progressively as function of the duration of preservation (6, 12, and 24 h) and all the parameters studied became significantly different from baseline after 24 h of the current static storage.

All procedures involving rats were conducted in accordance with internationally accepted principles for care of laboratory animals (EEC Council Directive 86/609, OJ L358,1, December 12, 1987) and the guidelines approved by the ethical committee of our University.

### 2.3. Analytical Methods

#### 2.3.1. Liver Ultrastructure

Liver samples were cut into small pieces, fixed in 4% formaldehyde solution and postfixed in 1% OsO_4_ in 0.1 M Sörensen buffer. All the samples were then dehydrated in alcohol and embedded in Epon. Thin sections were double-stained with uranium and lead and observed with a Philips 410 Electron Microscope.

#### 2.3.2. Hepatic Glycogen

PAS staining of liver tissue was performed to assess the glycogen stores. Briefly, the formalin-fixed and paraffin-embedded specimens were cut into slices of 4 *μ*m thickness and mounted on glass slides. Thin sections were deparaffinized, rehydrated in distilled water, and then pretreated for 5 min with 1% periodic acid (Fluka Chemie, Buchs, Switzerland). After washing in mQ-water, the section was left to react with the Schiff's reagent (Sigma-Aldrich, Milan, Italy) for 15 min at room temperature. Nuclei were counterstained for 2 min with Mayer's haematoxylin solution (Sigma-Aldrich, Milan, Italy).

#### 2.3.3. Hepatic Oxidative Stress

**GSH and GSSG Assay**

Liver specimens were homogenized in 10 volumes of 0.1 mol/L potassium-phosphate buffer (pH 7.4) containing 5 mmol/L EDTA. Enzymatic determination of total (GSH) and oxidized (GSSG) glutathione concentrations were performed by precipitating tissue homogenates with 15% sulfosalicylic acid (SSA) [16]. The supernatant was processed for GSH determination by the GSSG recycling procedure and incubated with 2-vinylpiridine and triethanolamine for GSSG assay [17].

**PSH and PSSG Assay**

Protein sulfhydryls (PSHs) were measured with a modification of Elmann's procedure in which the SSA-precipitated proteins were resuspended in 700 *μ*L of 6 M guanidine, pH 6.0. Optical density was read spectrophotometrically at 412 and 530 nm before and after 30 min of incubation with 50 *μ*L of 10 mM 5,5-dithiobis 2-nitrobenzoic acid [18]. Protein-glutathione mixed disulfides (PSSG) were measured as previously described [19]. Proteins were precipitated with 15% SSA containing 0.02 M EDTA and then dissolved in 300 *μ*L of 0.2 M ammonium bicarbonate containing 8 M urea and mixed with 5 mg Na2BH4. Pentanol (50 *μ*L) was added to avoid frothing. After 20 min, proteins were precipitated with 100 *μ*L of 15% SSA. The amount of GSH in the supernatant obtained after centrifugation at 45,000 g for 15 min was enzymatically measured [16]. Results are expressed as nmol GSH/mg protein.

**RSNO Assay**

The method described by Cook et al. [20], which uses a mixture of SULF/NEDD (sulfanilamide/N-(1-naphtyl)ethylenediamine dihydrochloride, neutral Griess) as reagents, was used. Liver homogenate was suspended 1 : 4 in PBS (pH 7.4) containing 10 mM N-ethylmaleimide and 4 mM potassium ferricyanide, acidified with 25% SSA, and centrifuged at 10,000 ×g for 10 min. The supernatant was added to 40 *μ*L of 1% ammonium sulfamate, 200 *μ*L of 0.4 N HCl containing 0.3% HgCl_2_ and 4.6% SULF, and 300 *μ*L of 0.4 N HCl containing 0.2% NEDD. After 30 min of incubation at 25°C, the samples were spectrophotometrically analyzed at 544 nm. Standards were prepared by reacting equal molar reduced glutathione and nitrite in water. Total protein concentration in liver homogenate did not differ significantly among the experimental groups.

### 2.4. Endothelial and Inducible Nitric Oxide Synthase (NOS) Gene Expression

The eNOS and iNOS gene expression was assessed by using real-time PCR. Briefly, total RNA was extracted from liver samples using Trizol/chloroform extraction (Invitrogen, Carlsbad, CA, USA). Total RNA quality was checked on ethidium bromide-stained gel and then 1 *μ*g reverse transcribed using SuperScript III RT (Invitrogen, Carlsbad, CA, USA). Oligonucleotide primers pairs for eNOS and iNOS were designed using Beacon Designer 2.0 software (BioRad, Richmond, CA, USA) and then purchased from Tema Ricerche s.r.l. (San Lazzaro, BO, Italy). As internal reference, primers for *β*-actin and *β*2-microglobulin were used. Real-time quantitative PCR experiments were performed utilizing iQSybr Green Supermix (BioRad, Richmond, CA, USA) in the iCycler (BioRad, Richmond, CA, USA) instrument. The fold changes in gene expression relative to the levels obtained in baseline control rats, which were considered equal to 1, were analyzed and calculated with the 2^−ΔΔCt^ method [21].

### 2.5. Statistical Analysis

Data were analyzed by means of the two-way analysis of variance (ANOVA) with Bonferroni's post hoc test. Statistical analysis was performed by the SPSS 8.0 statistical package. Data are reported as mean values ± SE. Two-tailed *P* values less than 0.05 were regarded as statistically significant.

## 3. Results

### 3.1. Liver Ultrastructure

**Figure 1:**
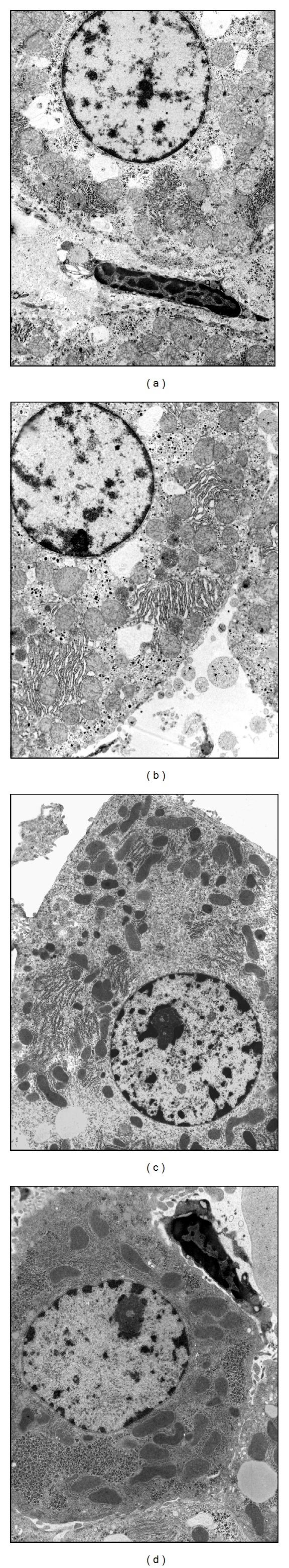
Transmission electron microscopy representative pictures of 24 h preserved livers. (a) Normobaric static preservation; (b) hyperbaric static preservation; (c) normobaric dynamic preservation; and (d) hyperbaric dynamic preservation.

After 24 h of the current static storage, hepatocytes showed many cytoplasmatic vacuoles and enlarged mitochondria with a reduced electron transparency and granular electron opaque aggregates (very likely composed by calcium salts) frequently found in the matrix. The nuclei presented extensive clumping of the chromatin and ribonucleoprotein components, while nucleoli were hardly visualized. Sinusoidal cells were detached and their nuclei were pycnotic (Figure 1(a)).

The ultrastructural alterations of hepatocytes and sinusoidal cells were clearly evident also in livers preserved under hyperbaric static conditions (Figure 1(b)). In contrast, normobaric dynamic preservation induced a significant improvement of mitochondria morphology, which appeared well preserved in most cases, even though some mitochondria presented moderate swelling. However, changes in the distribution of the nucleolar components and clumping of the nucleoplasm ribonucleoproteins were still present. Finally, the sinusoidal cells were detached from hepatocytes (Figure 1(c)).

Interestingly, hepatocytes of livers preserved with the combination of continuous perfusion and hyperbaric oxygen maintained an almost regular nuclear and cytoplasmatic ultrastructure. Nucleoli were easily recognized with a normal distribution of the ribonucleoproteins in most cases, while glycogen stores and mitochondrial membranes and matrix were well preserved, showing almost no matrix swelling and no membrane changes. Finally, sinusoidal cells appeared to be characterized by pycnotic nuclei (Figure 1(d)).

### 3.2. Hepatic Glycogen

**Figure 2:**
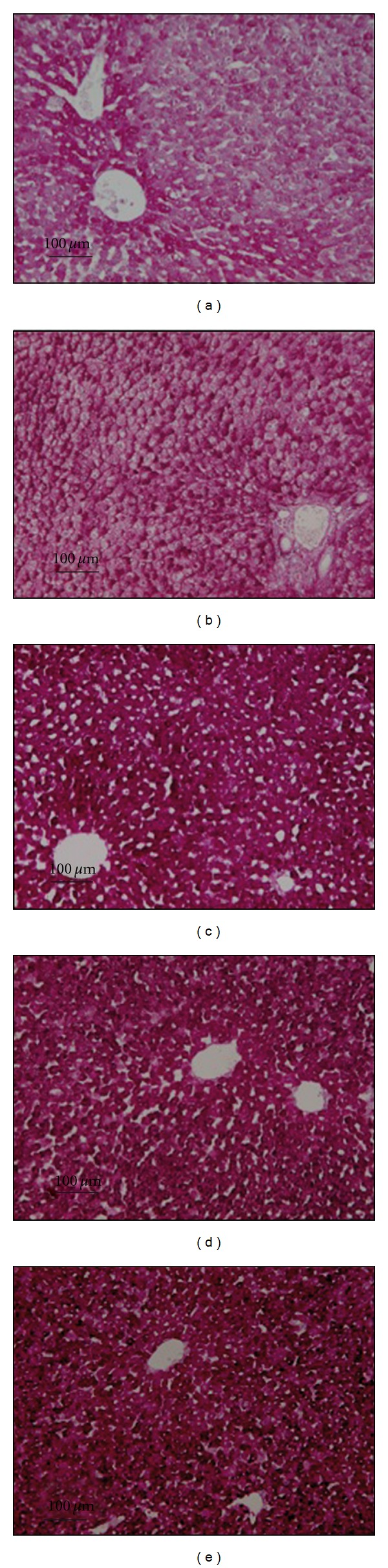
Representative histological pictures of the liver PAS-stained glycogen. (a) Normobaric static preservation; (b) hyperbaric static preservation; (c) normobaric dynamic preservation; (d) hyperbaric dynamic preservation; and (e) baseline controls.

Although the periodic acid Schiff staining allows only a qualitative evaluation, the hepatic glycogen content was clearly depleted after the current static storage (Figure 2(a)). While hyperbarism alone seems to have only a marginal effect (Figure 2(b)), dynamic preservation both with normobaric (Figure 2(c)) and hyperbaric oxygen (Figure 2(d)) appears to guarantee the maintenance of the baseline glycogen levels (Figure 2(e)).

### 3.3. Hepatic Oxidative Stress

**Figure 3:**
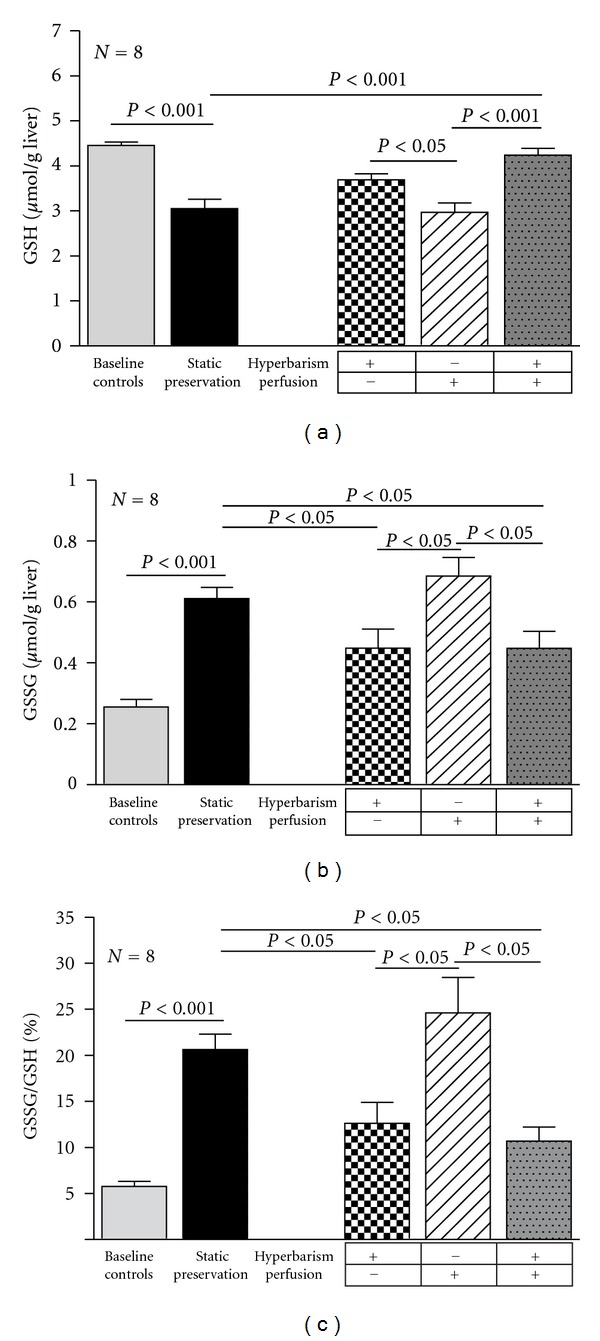
Total glutathione (GSH) concentration (a), oxidized glutathione (GSSG) concentration (b), and oxidized/total glutathione (GSSG/GSH) ratio (c) in baseline controls and 24 h preserved livers according to the study protocol. Data are expressed as means ± SE.

The tissue content of GSH, the major antioxidant in the liver, was significantly reduced after the current static storage as compared to the baseline level (Figure 3(a)). Taken into account the concomitant rise of GSSG, the oxidized form of glutathione (Figure 3(b)), one may conclude that the current static storage is associated with the occurrence of a significant oxidative stress, as shown by the increased GSSG/GSH ratio (Figure 3(c)). While normobaric dynamic preservation had no effect on these alterations, hyperbaric oxygen both under static and dynamic conditions partially counterbalanced the redox changes of the glutathione status.

**Figure 4:**
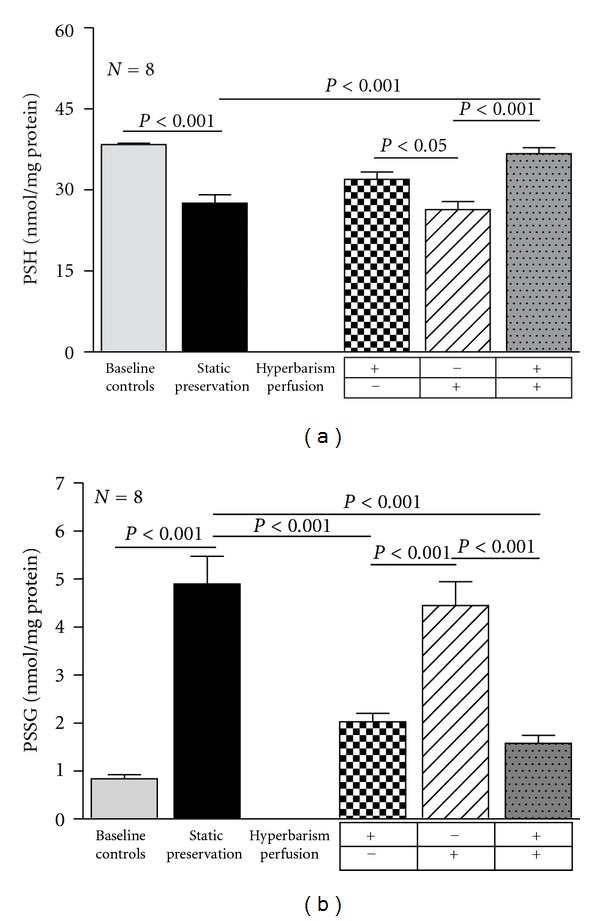
Protein sulfhydryls (PSH) concentration (a) and protein-glutathione mixed disulfides (PSSG) concentration (b) in baseline controls and 24 h preserved livers according to the study protocol. Data are expressed as means ± SE.

The changes of PSH and PSSG mirror those of GSH and GSSG (Figure 4).

**Figure 5:**
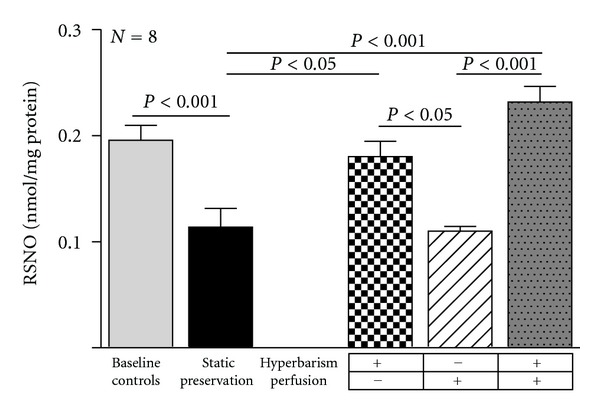
Nitrosothiols (RNSO) concentration in baseline controls and 24 h preserved livers according to the study protocol. Data are expressed as means ± SE.

Finally, the RSNO content, which represents an important quote of nitric oxide (NO) bound to free thiols, was reduced by 40% in livers preserved with the current static storage. Again, hyperbaric oxygen both under static and dynamic conditions prevented the RSNO depletion. In contrast, no effect was seen when continuous perfusion was performed under normobaric condition (Figure 5).

### 3.4. Hepatic Gene Expression of eNOS and iNOS

**Figure 6:**
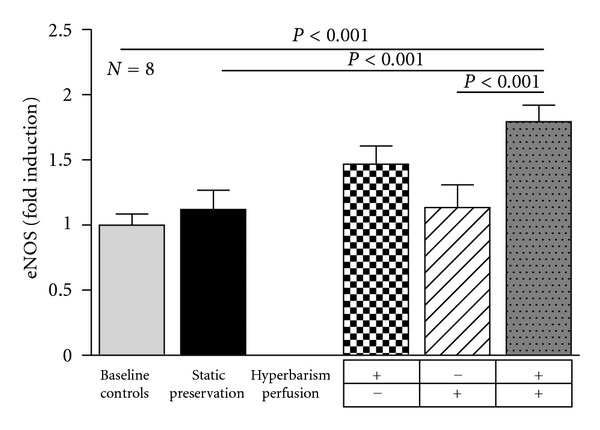
Endothelial nitric oxide synthase (eNOS) gene expression in baseline controls and 24 h preserved livers according to the study protocol. Values were determined by real-time PCR and expressed as fold induction over baseline control rats. Data are expressed as means ± SE.

The basal preischemic gene expression of eNOS remained substantially unchanged in the livers maintained for 24 h under the current static cold storage. While perfusion alone had no evident effect, hyperbaric preservation was associated with an increased eNOS gene expression although a statistical significant difference was reached only in the group combining continuous perfusion and hyperbaric oxygenation (Figure 6). In contrast, the gene expression of the iNOS isoform after 24 h preservation was similar in all the experimental groups to that observed in basal preischemic livers (data not shown).

## 4. Discussion

The HMP has been shown to challenge the limits of the current static storage in experimental liver transplantation, by providing continuous nutrients and oxygen supply to the organ and by removing the waste products, thus improving parenchymal cell viability and function even after prolonged cold ischemia [4, 9]. More recently, a pilot study has also shown the safety and feasibility of liver preservation with HMP in 20 liver transplanted patients [22, 23]. Although active oxygenation of the preservation solution was not provided by the HMP used in this study, the pO_2_ levels in the effluent perfusate remained relatively high and stable during preservation as a result of ambient air interchange at the organ chamber [22].

The hyperbaric hypothermic machine perfusion (HHMP) described in our study represents an innovative approach against liver preservation injury, which combines the oxygenation of the preservation solution with hyperbaric oxygen to the continuous graft perfusion as performed by the actual HMPs. We found that hyperbaric oxygen, besides maintaining the positive effects of continuous perfusion on ultrastructural damage and glycogen depletion, produces an extra benefit by reducing the oxidative injury occurring during preservation under normobaric conditions and by inducing eNOS gene expression.

In these experiments, a significant oxidative damage occurred after 24 h of static or dynamic normobaric preservation, as indicated by the increased oxidized glutathione and the GSSG/GSH ratio and by the reduced PSH. The enhanced production of ROS during cold ischemia has been already reported [24, 25] and only apparently it represents a paradox. Indeed, the decrease of O_2_ concentration to the hypoxic range of 5 to 0.5% [26] would lower mitochondrial respiration and enhance the reduction state of the mitochondrial redox centres, favouring the electron leak to molecular oxygen and ROS production, mainly by the respiratory complexes I and III [27]. Thus, oxygen acts as a double-edged sword during cold preservation: its supply is mandatory to support aerobic metabolism and maintain ATP levels in the hepatocytes, but it is also potentially harmful by increasing the generation of toxic ROS.

In contrast, preservation with hyperbaric oxygen was associated with decreased oxidative injury. Although previous studies have shown that hyperbaric oxygen reduce lipid peroxidation in several prooxidant experimental models [12, 28, 29], the underlying mechanisms of protection are still elusive. It can be hypothesized that hyperbaric oxygen favours an efficient electron transfer through the OXPHPOS complexes, as suggested by the great improvement of ultrastructural mitochondrial damage observed in a previous work [30], and reduces ROS formation, which eventually may prevent the GSH oxidative consumption.

However, under static conditions, hyperbaric oxygen reduces oxidative damage without improving mitochondrial damage, thus suggesting the existence of other mechanisms. Nitrosothiols are unstable thioesters exerting different intra- and extracellular functions only in part defined [31]. Recent experimental evidences suggest that protein S-nitrosylation could modulate ROS production and limit oxidative damage during ischemia reperfusion in the heart and liver [32]. In our experiments, liver preserved under hyperbaric conditions maintain the baseline nitrosothiols levels, which are instead significantly reduced after normobaric preservation. Since protein S-nitrosylation has emerged as an effector of NO bioactivity [33], it is also tempting to speculate that the prevention of nitrosothiols depletion may be related to an increased generation of NO from eNOS, as suggested by the induction of its gene expression in livers preserved under hyperbaric conditions.

Beside the effect on protein S-nitrosylation and oxidative stress, a greater availability in the vascular bed of eNOS generated NO may have other potential benefits by favouring vasodilatation and capillary flow and by reducing neutrophil adherence during reperfusion of the graft [13–15]. As we were not able to observe any significant effect on iNOS gene expression, our results support the finding of previous studies that hyperbaric oxygen is able to modulate NO production in several organs and different experimental conditions by selectively inducing eNOS while inhibiting iNOS production [13–15].

Although it can be expected that the prevention during cold preservation of mitochondrial damage, glycogen depletion, and oxidative injury, together with the increased endothelial NO generation, can favour a rapid recovery of liver graft viability after reperfusion, whether the use of HHMP can effectively improve the post-transplant outcome compared to the current static storage remains undetermined and needs to be assessed in experimental liver transplantation, which was not technically feasible in the present study. Besides that, several other aspects have to be tested in order to optimize the use of the HHMP, including the type of preservation solution, the rate and modalities of organ perfusion, and the degree of hyperbarism. In the present study, we decided to minimize the number of the experimental variables in order to selectively assess the effect of hyperbarism combined or not with the continuous perfusion of the liver. With this objective in mind, we have utilized a preservation solution currently employed in the static cold storage as well as an intermediate rate of perfusion and a medium level of hyperbarism among those employed in the studies previously published [4, 7, 9, 15, 22, 34]. Of course, it means that the use of preservation solutions specifically designed for machine perfusion systems or different perfusion dynamics would likely produce some additional benefit.

A potential pitfall associated with the continuous perfusion of the graft is represented by the sinusoidal endothelial cell (SEC) damage, which likely ensues, beside the well-established detrimental effects of hypothermia, from a shear-stress phenomenon [9, 35], thus counterbalancing the potential benefits on cell metabolism. Several attempts to improve SEC viability are under investigation, including modifications in the composition of the preservation solutions and in the modalities of organ perfusion [7, 9, 34, 36]. In our experiments, while continuous perfusion with Celsior solution at 1 mL/min/g liver with hyperbaric oxygen did not appear to be fully protective, we found that lowering the rate of perfusion flow to 0.5–0.2 mL/min/grams of liver was associated with a better preservation of the sinusoidal lining (data not shown).

In conclusion, preservation by means of the present innovative HHMP, which combines the oxygenation of the preservation solution with hyperbaric oxygen to the continuous graft perfusion, reduced liver injury possibly by preventing the mitochondrial damage, glycogen depletion and oxidative damage which develop after the currently used static storage. Interestingly, only the use of hyperbaric oxygen was associated to a blunted oxidative stress and an increased eNOS gene expression. These results represent the rationale background to test the HHMP in experimental liver transplantation models.

## Abbreviations

GSH: Total glutathione
GSSG: Oxidized glutathione
HMP: Hyperbaric Machine Perfusion
HHMP: Hypothermic Hyperbaric Machine Perfusion
NO: Nitric oxide
eNOS: Endothelial nitric oxide synthase
OXPHOS: Oxidative phosphorylation
PSH: Protein sulfhydryls
PSSG: Protein-glutathione mixed disulfides
RSNO: Nitrosothiols.
